# Quantitative Analysis of Long-Form Aromatase mRNA in the Male and Female Rat Brain

**DOI:** 10.1371/journal.pone.0100628

**Published:** 2014-07-18

**Authors:** Nino Tabatadze, Satoru M. Sato, Catherine S. Woolley

**Affiliations:** Department of Neurobiology, Northwestern University, Evanston, Illinois, United States of America; John Hopkins University School of Medicine, United States of America

## Abstract

In vitro studies show that estrogens acutely modulate synaptic function in both sexes. These acute effects may be mediated in vivo by estrogens synthesized within the brain, which could fluctuate more rapidly than circulating estrogens. For this to be the case, brain regions that respond acutely to estrogens should be capable of synthesizing them. To investigate this question, we used quantitative real-time PCR to measure expression of mRNA for the estrogen-synthesizing enzyme, aromatase, in different brain regions of male and female rats. Importantly, because brain aromatase exists in two forms, a long form with aromatase activity and a short form with unknown function, we targeted a sequence found exclusively in long-form aromatase. With this approach, we found highest expression of aromatase mRNA in the amygdala followed closely by the bed nucleus of the stria terminalis (BNST) and preoptic area (POA); we found moderate levels of aromatase mRNA in the dorsal hippocampus and cingulate cortex; and aromatase mRNA was detectable in brainstem and cerebellum, but levels were very low. In the amygdala, gonadal/hormonal status regulated aromatase expression in both sexes; in the BNST and POA, castration of males down-regulated aromatase, whereas there was no effect of estradiol in ovariectomized females. In the dorsal hippocampus and cingulate cortex, there were no differences in aromatase levels between males and females or effects of gonadal/hormonal status. These findings demonstrate that long-form aromatase is expressed in brain regions that respond acutely to estrogens, such as the dorsal hippocampus, and that gonadal/hormonal regulation of aromatase differs among different brain regions.

## Introduction

Cytochrome P450 aromatase, the product of the CYP19 gene, catalyzes the biosynthesis of estrogens from androgens. Multiple peripheral tissues including ovaries and testes, placenta, adipose tissue, bone, and muscle express aromatase and hence could synthesize estrogens (reviewed in [1]). In addition, aromatase activity has been detected in the brains of both male and female rats, originally shown by Naftolin and colleagues [2]. Since this early work, numerous studies have investigated aromatase expression and/or activity in the central nervous system of multiple vertebrate species [3]–[6], including humans [7]–[8]. High levels of aromatase activity are found in brain regions involved in reproductive neuroendocrine functions, including in the medial basal hypothalamus, bed nucleus of stria terminalis (BNST), preoptic area (POA) and amygdala [4], [9]. Interestingly, aromatase has also been reported in brain regions not closely linked to reproduction, such as the hippocampus and cerebral cortex [10]–[12]. This suggests that, in addition to reproductive functions, aromatase may play a role in modulation of mood, affective behaviors and/or learning and memory, which are supported by these non-reproductive brain areas.

Although the classical mode of estrogen action is through nuclear receptors that regulate gene expression, estrogens also influence neurophysiology acutely, on a timescale of minutes, which is too rapid to require gene expression (reviewed in [13]). For example in the hippocampus, estradiol (E2) potentiates excitatory synaptic transmission [14]–[17] and suppresses inhibitory synaptic transmission [18] on a timescale of minutes. Ovarian estrogens are unlikely to be the physiological ligands that activate these acute effects *in vivo*, as circulating estrogens fluctuate on a timescale of days. An alternative idea is that aromatase within brain areas such as the hippocampus generates local and possibly rapid fluctuations in estrogens that acutely modulate synaptic function.

For this to be the case, however, aromatase expressed in the hippocampus (or other brain regions) must be capable of synthesizing estrogens. Studies using a ribonuclease protection assay demonstrated that there are two forms of aromatase mRNA in the rat brain: a long form and a short form, and suggested that the long form generates the active enzyme [19]. Among the brain regions that were examined, expression of long-form aromatase exhibited regional differences that correlated with aromatase activity, whereas short-form did not. These initial observations were confirmed using an RNA probe that targeted the long form of aromatase specifically [10], [20]. The presence of two forms of aromatase may explain mismatch in the distribution of aromatase protein in the brain detected by immunohistochemistry [21]–[23] and aromatase activity measured by enzyme assays [5], [19].

To investigate the distribution of aromatase in the rat brain, and avoid caveats of antibody specificity, we used quantitative real-time PCR (qPCR) to measure relative mRNA levels specifically of long-form aromatase in the amygdala, BNST, POA, dorsal hippocampus, cingulate cortex, brainstem and cerebellum in both male and female rats. We also evaluated possible hormonal regulation of long-form aromatase in each sex. We were particularly interested to determine whether the female rat hippocampus expresses long-form aromatase, as this has not been shown previously, yet is predicted by the observation that E2 acutely modulates synaptic transmission in the hippocampus of females.

## Methods

### Animals

All animal procedures were performed in accordance with the National Institutes of Health *Guide for the Care and Use of Laboratory Animals* and were approved by Northwestern University Animal Care and Use Committee (protocol numbers: 2011-2521-5 and 2011-1400-7). All animals were young adult Sprague Dawley rats (50–60 days old; Harlan). Six gonadally intact females were used to assess fluctuation in ovarian aromatase expression across the estrous cycle. In these intact females, estrous cycle stages were monitored for at least 5 consecutive days and ovaries were harvested from 2 rats each in proestrus, estrus, and diestrus. The remaining 9 males and 9 females were divided into the following groups: castrated males (n = 4); gonadally intact (sham castrated) males (n = 5); ovariectomized (ovx) females (n = 4); and ovariectomized/estradiol (E2)-treated (ovx+E2) females (n = 5). All gonadectomies were carried out under general anesthesia (ketamine, 85mg/kg; xylazine, 13 mg/kg, i.p.) using aseptic surgical protocol. For ovx, ovaries were removed through bilateral incisions on the dorsal flank after blood vessels were ligated. Ovariectomy of females was performed in diestrus and ovaries were collected during the surgery, frozen immediately on dry ice and stored at −80°C for later analysis. For castration, testes were removed through a single incision along the midline in the scrotum after blood vessels were ligated. For sham castration, the incision was made and closed without removal of testes. Animals were given one week to recover after surgery. Seventy-two and 48 hours prior to sacrifice, female rats in the E2-treated group were given two s.c. injections of 10 µg 17β-estradiol benzoate in 100 µl sesame oil vehicle. This dose of estradiol benzoate produces supraphysiological estradiol levels shortly after injection, which are metabolized to within the physiological range by 24 hours later [24]. All other groups were given 100 µl injections of vehicle alone.

### Tissue collection

All tissue collection was carried out under RNase-free conditions. At the time of sacrifice, rats were deeply anesthetized with sodium pentobarbital (80 mg/kg, i.p.; Virbac Animal Health), and then tissue from peripheral organs (liver, lung, spleen, and skeletal muscle), the cerebellum, and brainstem (BS) was collected from each animal, pooled within group into a 1.5 ml RNase-free tube, and snap frozen on dry ice. The rest of the brain from each animal was frozen on dry ice and stored at −80°C. Frozen brains were then individually mounted in a cryostat, warmed to −4°C and 500 µm coronal sections were cut. Tissue samples from the preoptic area (POA), amygdala (Amyg), bed nucleus of the stria terminalis (BNST), dorsal hippocampus (dHipp), and cingulate cortex (CC) were punch dissected from these coronal sections using either 12 ga (2.4 mm, ID) or 15 ga (1.6 mm, ID) punches, and the tissue from the same region within each group was pooled into 1.5 ml RNase-free tubes. The tubes first were kept on dry ice to prevent thawing of the tissue and subsequently were stored at −80°C for later analysis.

### RNA isolation

Tissue samples were thawed in 1 ml TRIzol Reagant (Ambion) and homogenized with a tissue sonicator (Qsonica) using three 3 sec pulses. Samples were centrifuged at 12,000 x g for 10 min at 4°C and then the supernatant was carefully removed and transferred to a new tube. After 5 min at room temperature, 0.2 ml chloroform was added; tissue was mixed vigorously, allowed to stand at room temperature for 3 min before centrifugation at 12,000 x g for 15 min at 4°C. The aqueous phase was transferred to a new tube, and total RNA was isolated and purified using Purelink RNA Mini Kit (Ambion) according to the manufacturer's instructions. RNA was eluted with 30 µl RNase-free water. RNA concentration was measured using a spectrophotometer (Nanodrop, Thermo Scientific) at a wavelength of 260 nm and RNA purity was assessed based on an A260/A280 ratio of 1.8–2.0.

### Reverse transcription

Reverse transcription reactions were performed using the GoScript Reverse Transcription System (Promega). RNA (2 µg) was mixed with 1 µl Oligo(dT)_15_ Primer, 1 µl random primers and nuclease-free water for a total volume of 5 µl. Samples were incubated in a heat block at 70°C for 5 min followed by immediate chilling on ice for 5 min. The reverse transcription master mix for each sample consisted of 4 µl GoScript 5x reaction buffer, 3 µl MgCl_2_, 1 µl PCR nucleotide mix, 0.5 µl recombinant RNasin (ribonuclease inhibitor), 1 µl GoScript reverse transcriptase, and 5.5 µl nuclease-free water. The master mix was added to each sample for a total reaction volume of 20 µL. Samples were incubated at 25°C for 5 min (annealing), 42°C for 1 hr (extending), and 70°C for 15 min (reverse transcriptase inactivation). After the reaction was completed, samples were spun down in a microfuge for 5 sec and stored at −20°C.

### Quantitative real-time PCR (qPCR)

**Figure 1:**
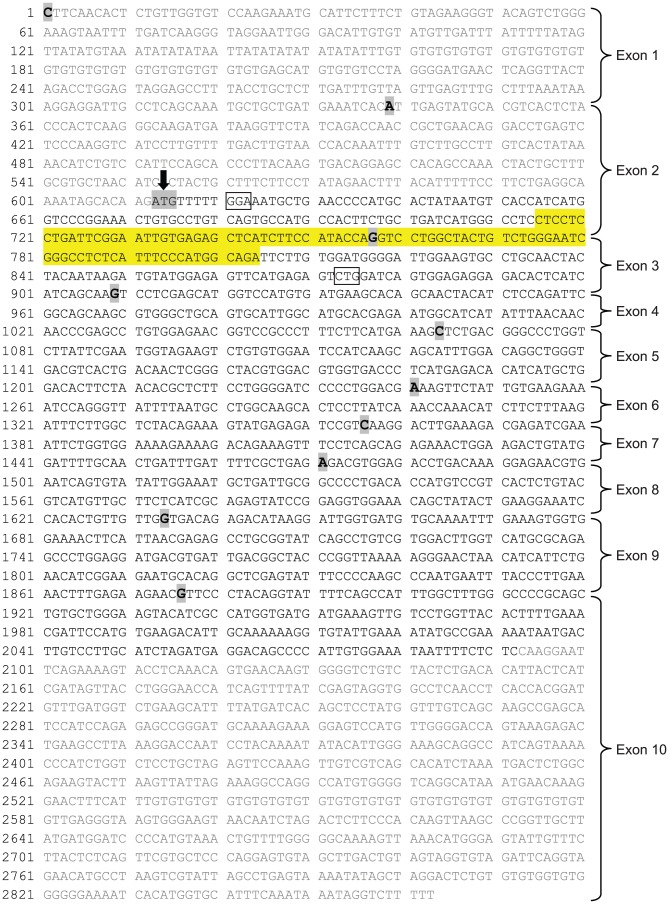
mRNA sequence of rat aromatase. Exon boundaries in the rat mRNA sequence of cytochrome P450 (NCBI Reference Sequence: NM_017085.2) are marked in bold and highlighted in grey. An arrow indicates beginning of the translated region in the long form of aromatase. Untranslated regions have been grayed out. The 90 bp fragment (715–805) that was amplified in our qPCR and RT-PCR analyses is highlighted in yellow. The region that is thought to be missing in the truncated form of the enzyme involves the exons 2 and 3 and hence encompasses the sequence that was targeted in our study. The boxed nucleotides in the sequence indicate the start and the end of the RNA probe that protected only the long form of aromatase in ribonuclease protection assays [10].

qPCR was performed using SYBR green to determine transcript levels for aromatase and 3 reference genes: glyceraldehyde 3-phosphate dehydrogenase (GAPDH), hypoxanthine phosphoribosyltransferase (HPRT) and succinate dehydrogenase complex, subunit A (SDHA). Target sequences were amplified using the following primer pairs: aromatase (F) 5′-CTCCTCCTGATTCGGAATTGT-3′ and (R) 5′-TCTGCCATGGGAAATGAGAG-3′; GAPDH (F) 5′-GGGTGTGAACCACGAGAAATA-3′ and (R) 5′-AGTTGTCATGGATGACCTTGG-3′; HPRT (F) 5′-GGCCAGACTTTGTTGGATTTG-3′ and (R) 5′-CTTTCGCTGATGACACAAACAT-3′, and SDHA (F) 5′-ATGGAAAATGGGGAGTGCCG-3′ and (R) 5′-GCTGAAGTAGGTTCGCCCAT-3′. The primer pair efficiencies were determined based on the slope of standard curve that was generated after plotting the results of the titration of the target cDNA (C_T_ values vs log [dilution] within the range of linear amplification. The efficiencies were as follows: aromatase –99.6% (slope: −3.235), GAPDH –99.8% (slope: −3.3), HPRT –100% (slope: −3.39) and SDHA –97.8% (slope: −3.376). The primer pair for aromatase targeted a region that contains parts of exons 2 and 3, which is present only in long-form aromatase. The PCR product was a 90 bp fragment (shown in Figure 1) within the region that was targeted by an RNA probe that protected only the long form of aromatase in ribonuclease protection assays [10]. Based on comparison of aromatase expression in peripheral tissues, ovary was used as a positive control and liver was used as a negative control (see Results).

The PCR reactions were prepared in triplicate in a 384-well plate by mixing 1.2 µl cDNA (10 µg) and 1.2 µl commercially available GoTaq qPCR master mix (Promega) using a Mosquito LCP automated liquid handler (TTP Labtech) and then adding 10 nl primer mix (for 200 nM of each primer in the final reaction) with an Echo Liquid Handler (Labcyte). qPCR was carried out using the following program: 95°C for 5 min, 40 cycles of 95°C for 15 sec and 60°C for 1 min, and a melt curve analysis (95°C for 15 sec, 60°C to 95°C with 0.5°C intervals, 30 sec per interval). The melt curve was used to confirm the specificity of each PCR reaction. Relative gene expression was determined using the 2**^–ΔΔ(C^**_T_^**)**^ method (C_T_ = cycle threshold), where ΔΔC_T_  =  ΔC_T_ (sample) – ΔC_T_ [25]. The sample and reference data points were normalized to the geometric mean of all three housekeeping genes (GAPDH, HPRT, SDHA). The use of multiple housekeeping genes provides a more reliable control for qPCR [26], as the expression of individual genes may be affected by an experimental treatment [27]–[28].

Gene expression data were obtained from 4 qPCR runs per group using cDNA products of 2 independent reverse transcription reactions for each group. Each qPCR run was treated as an independent experiment. Mean aromatase mRNA levels were normalized to the levels of the housekeeping genes as described above, and used for statistical analysis (n = 4). Results were analyzed statistically using either a one-way ANOVA (estrous cycle stages) followed by Tukey's multiple comparison, or two-way ANOVA (sex x gonad/hormone condition), followed by independent-sample *t*-tests with Bonferroni's correction for *post hoc* analysis when applicable. p<0.05 was considered statistically significant.

### Reverse transcription PCR (RT-PCR) and gel electrophoresis

RT-PCR and gel electrophoresis was used to visualize the amplified aromatase cDNA fragment for each brain region and thereby corroborate qPCR results. Based on the differences in C_T_ values among different brain regions in qPCR analysis, the cycle number for each region was calculated using the average C_T_ value for that region and adding 6 cycles, which was the earliest stage of amplification in which DNA amplification products were reliably detectable on a gel. We used the same aromatase and GAPDH primer pairs for RT-PCR as in the qPCR analysis. The PCR reaction (in duplicates) consisted of 1 µl cDNA, 1.25 µl forward primer (10 µM), 1.25 µl reverse primer (10 µM), 12.5 µl GoTaq colorless master mix (Promega) and 9 µl nuclease-free water for a total reaction volume of 25 µl. The optimal hot-start PCR program was as follows: 95°C for 15 sec, 60°C for 1 min and 72°C for 4 min, with a final hold at 4°C. A 2% agarose gel was prepared using TAE buffer, and ethidium bromide was added before gel casting. DNA loading dye was mixed with the PCR products and loaded on the gel. The gel was run at 120V for 1 h and viewed with a gel imager (Kodak).

## Results

### Differential expression of aromatase among brain regions

**Figure 2:**
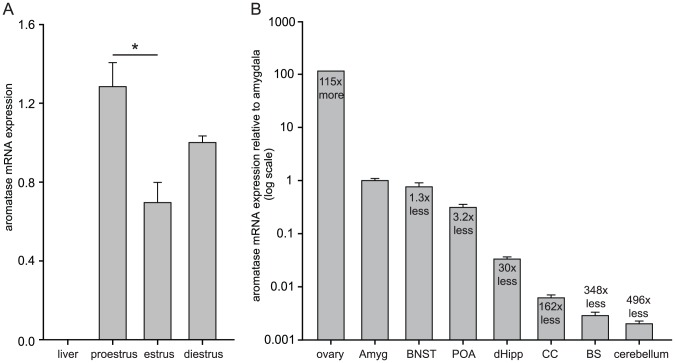
Differential expression of rat aromatase. **(A)** qPCR measurements of aromatase mRNA levels in the ovary at different phases of estrous cycle are shown. Ovarian aromatase levels were highest in proestrus, intermediate in diestrus, and lowest in estrus. Liver was used as a negative control. *significant difference between proestrus and estrus (p<0.05). **(B)** qPCR measurements of aromatase mRNA levels in diestrus ovary compared to multiple brain regions: amygdala, bed nucleus of the stria terminalis (BNST), preoptic area (POA), dorsal hippocampus (dHipp), cingulate cortex (CC), brainstem (BS), and cerebellum. For each brain region, data from four groups of rats (gonadally intact male, castrated male, ovx female and ovx+E2 female) were averaged. Values are expressed relative to the amygdala, set as 1.0, and fold-differences in expression are relative to amygdala.

Because aromatase is expressed broadly, we first sought to identify reliable positive and negative controls. Among the peripheral tissues that we tested (liver, lung, spleen, skeletal muscle), liver showed no detectible aromatase mRNA and hence was used as a negative control. Ovary was chosen as a positive control for its high aromatase expression [29]. Given that E2 levels fluctuate throughout the reproductive cycle, it is expected that ovarian aromatase expression also varies across the cycle [30]. Analysis of aromatase mRNA levels in ovaries from rats in different estrous cycle stages showed that aromatase expression was highest in proestrus, lowest in estrus, and intermediate in diestrus [F(2,3) = 9.82, p = 0.048, Figure 2A] with a statistically significant difference between proestrus and estrus (p<0.05). mRNA isolated from ovaries in diestrus was used in all subsequent qPCR experiments for comparison with brain regions.

Next, we carried out qPCR analyses of aromatase expression in different brain regions of rats from 4 groups: castrated males, gonadally intact males, ovx females and ovx+E2 females. Because the range of aromatase expression between brain areas was much larger than group differences within an area, we first plotted the mean aromatase expression for all 4 groups in each region (Figure 2B). Of all brain regions tested, average aromatase expression was highest in the amygdala, which was 115 times lower than in ovary. Aromatase expression in the amygdala was then set as 1.0 and levels in other brain regions were plotted relative to amygdala. This showed that areas involved in reproductive functions have the highest aromatase mRNA expression: amygdala (1.0±0.092, mean C_T_ = 22) was followed by BNST (0.76±0.142, mean C_T_ = 23, 1.3 times lower than amygdala) and POA (0.311±0.044, mean C_T_ = 24, 3.2 times lower than amygdala). Cortical structures exhibited intermediate aromatase mRNA expression; higher in dorsal hippocampus (0.033±3.38E-3, mean C_T_ = 27, 30 times lower than amygdala) than in cingulate cortex (0.0062±8.63E-4, mean C_T_ = 30, 162 times lower than amygdala). The two other brain areas examined, brainstem (2.87E-3±4.47E mean C_T_ = 33,) and cerebellum (2.01E-3±2.61E-4 mean C_T_ = 35,), showed much lower aromatase expression (348 times and 496 times lower than amygdala, respectively).

### Sex and gonadal effects on aromatase expression in different brain regions

To determine the contributions of sex and hormonal/gonadal status on aromatase expression, aromatase mRNA levels within each brain region were compared between castrated males, gonadally intact males, ovx females, and ovx+E2 females. This showed 3 distinct patterns of regulation; 1) both sex and gonadal hormones affect aromatase expression (amygdala), 2) gonadal hormones up-regulate aromatase mRNA expression only in males (BNST and POA), or 3) no differences in aromatase expression regardless of sex or hormonal/gonadal status (dorsal hippocampus and cingulate cortex). Aromatase mRNA levels were too low in the brainstem and cerebellum for meaningful comparisons between groups. For quantitative comparisons between groups, the mean aromatase mRNA expression value of ovx females was set as 1.0 and data for the other groups were expressed relative to ovx females.

**Figure 3:**
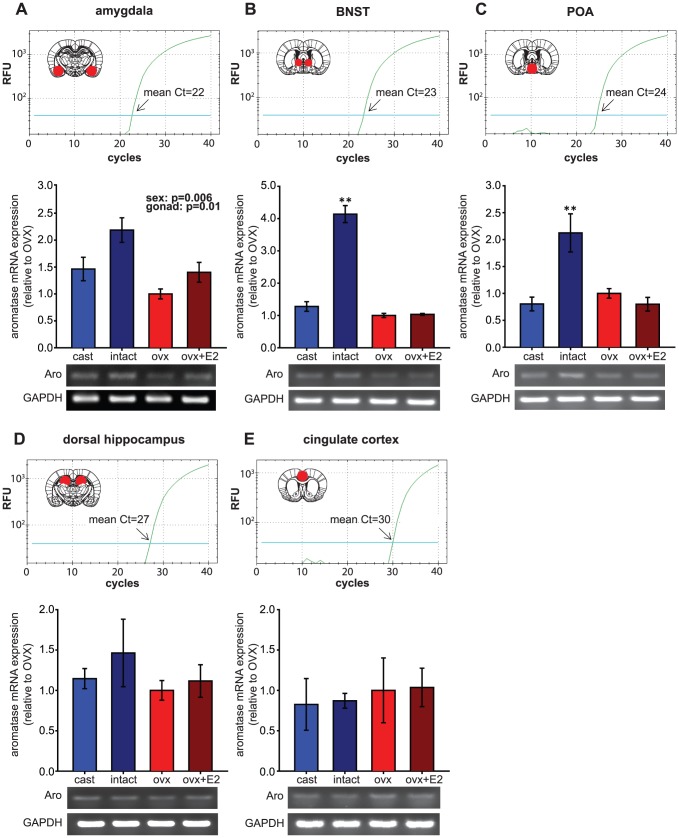
Effect of gonadal/hormonal status on aromatase mRNA expression in different brain regions. Expression levels of the long form of aromatase were studied in the amygdala, BNST, POA, dorsal hippocampus, and cingulate cortex of castrated male, gonadally intact male, ovx female and ovx+E2 female rats. Each panel represents a different brain region. For each panel, qPCR amplification curves with relative fluorescent units (RFU) and the area punch dissected (red circle in the schematics of brain coronal sections, adapted from [48]) are shown at the top. C_T_ (cycle threshold) values on the amplification curve indicate the number of cycles required for the fluorescent signal to cross the threshold, which was set at 10 standard deviations above mean fluorescence generated during baseline cycles. Relative aromatase mRNA expression for each brain region is shown in bar graphs (ovx females set as 1.0) with representative images of agarose gels showing RT-PCR products for amplified aromatase (Aro) and GAPDH DNA fragments at the bottom. In the amygdala **(A)**, aromatase mRNA levels were affected by both sex (p = 0.006) and gonadal/hormonal status (p = 0.01), whereas in the BNST **(B)** and the POA **(C)**, gonadally intact males showed the highest aromatase mRNA expression, which was significantly different from all other groups; ** significant difference between intact males and other groups (p<0.01). In contrast, in the dorsal hippocampus **(D)** and cingulate cortex **(E)** aromatase mRNA levels did not differ among the groups.

In the amygdala, which showed the highest aromatase expression among the brain areas we examined, aromatase mRNA levels were higher in males by 52% compared to females [sex: F(1,12)  = 10.98, p  = 0.006, Figure 3A]. Furthermore, gonadal hormones up-regulated aromatase expression in both sexes [gonad: F(1,12)  = 8.92, p  = 0.01], by 40% (females) and 49% (males). The sex difference and the upregulation by gonad/hormone were additive, so that gonadally intact males showed the highest aromatase expression, followed by intermediate levels in castrated males and ovx+E2 females, and the lowest levels in ovx females. To corroborate qPCR results, we amplified the aromatase DNA fragment with RT-PCR and visualized the product on a gel. Figure 3A (bottom) shows the highest aromatase band intensity for gonadally intact males, and the lowest aromatase band intensity for ovx females with no differences in GAPDH.

In the BNST (Figure 3B) and the POA (Figure 3C), unlike the amygdala, hormonally driven up-regulation of aromatase mRNA was observed only in males [sex x gonad interaction: F(1,12)  = 82.94, p<0.001 in BNST, and F(1,12)  = 13.94, p = 0.003 in POA]. In the BNST of gonadally intact males, aromatase expression was 224% higher than in castrated males, and castrated males showed expression levels nearly identical to both groups of females. Similarly, in the POA of gonadally intact males, aromatase expression was 165% higher than in castrated males, which also showed expression similar to females. In both brain regions, that intact males had the highest aromatase expression was readily observable in the aromatase RT-PCR gels (BNST: Figure 3B, bottom, and POA: Figure 3C, bottom).

In contrast to the amygdala, BNST, and POA, aromatase expression in the dorsal hippocampus (Figure 3D) and cingulate cortex (Figure 3E) was not significantly influenced by sex or hormonal/gonadal status in either sex, which was corroborated by the lack of apparent differences in RT-PCR products (dHipp: Figure 3D, bottom, and CC: Figure 3E, bottom). The relative aromatase mRNA expression in the dorsal hippocampus followed a pattern similar to the amygdala (i.e. higher in males than in females and reduced by gonadectomy), but differences were much smaller in magnitude and not statistically significant.

## Discussion

The present study investigated relative expression of mRNA for the long form of aromatase in distinct regions of the male and female rat brain and the effect of gonadal/hormonal status on aromatase expression in both sexes. We focused on the long form of aromatase based on previous evidence that this is the catalytically active enzyme. Using qPCR, we found highest aromatase expression in the amygdala followed closely by the BNST and the POA, then the dorsal hippocampus and cingulate cortex, and much lower expression in the brainstem and cerebellum. This pattern was true for all groups regardless of sex or gonadal/hormonal status. The amygdala was the only brain region in which gonadal/hormonal upregulation of aromatase expression occurred in both sexes, though these effects were moderate in each sex. In the BNST and POA, castration had a strong effect in males to reduce aromatase expression to a level similar to that in females, whereas there was no evidence for estrogenic regulation in females. In the hippocampus and cingulate cortex, there were no significant differences in aromatase expression between sexes or gonadal/hormonal conditions. That the long form of aromatase is expressed in the dorsal hippocampus, including in females, supports the idea that estrogens synthesized within the hippocampus could acutely modulate synaptic transmission *in vivo* in both sexes.

### Relative levels of aromatase expressed in brain

Multiple previous studies in rodents have investigated aromatase levels in the brain, however few have taken into account evidence that aromatase exists in two forms of which the long form is the active one [10], [19]. One exception is Hojo et al. [11] who carried out RT-PCR analysis of aromatase mRNA expression in the gonadally intact male rat brain using primers that targeted long-form aromatase specifically. Although their findings on regional differences in aromatase expression are generally consistent with ours, there are also some differences, mainly in the magnitude of relative expression between each brain region and the ovary. Hojo et al. [11] reported that aromatase mRNA levels in the hippocampus are 300 times lower than in ovary and 500 times lower in the cortex compared to ovary. In the present study, we found that differences between the brain and ovary were greater. For example, our results indicate that aromatase mRNA levels in the dorsal hippocampus and cingulate cortex of gonadally intact males are 6600 and 25000 times lower than in ovary, respectively. These differences may be explained by the different PCR techniques used (qPCR vs RT-PCR), specificity of brain regions tested (dorsal vs entire hippocampus) and/or different estrous cycle stages that were used for comparison (diestrus vs unspecified).

### Regulation of aromatase expression by gonadal/hormonal status

The aromatase gene contains both androgen and estrogen response elements [31]–[33] suggesting aromatase gene regulation through classical steroid receptor-dependent mechanisms. Consistent with this, the brain regions in which we found gonadal/hormonal regulation of aromatase are rich in nuclear androgen and estrogen receptors [34]–[35].

Regulation of brain aromatase by gonadal hormones in rodents has been demonstrated previously, although most of these studies included only one sex and/or focused on brain regions involved in reproductive function. Our findings that expression of long-form aromatase in the BNST and POA of males is upregulated by the testis are consistent with previous studies in which castration of adult male rats decreased aromatase activity in hypothalamus-preoptic area and administration of testosterone or dihydrotestosterone prevented this decrease [19], [36]–[37]. In contrast to the BNST and POA, we found that the effect of the testis was less robust in the amygdala. That aromatase in the amygdala is less affected by hormones [19] may be related to differential hormonal sensitivity of amygdala subregions. For example, microdissection studies suggest that androgens regulate aromatase in the medial but not cortical amygdala [38].

In contrast to the role of androgens, less is known about how estrogens influence brain aromatase activity or gene expression. Several groups have reported estrogenic upregulation of aromatase expression in the POA, medial basal hypothalamus or amygdala in castrated or intact males [37], [39], though these studies did not distinguish between long and short forms of aromatase. In our study, E2 treatment of ovx female rats increased expression of the long form of aromatase in the amygdala, but was without effect in other brain regions tested. Iivonen et al. [40] used qPCR in the hippocampus of female mice and reported bidirectional regulation of aromatase mRNA expression (downregulation vs upregulation) depending on a phasic vs tonic E2 treatment regimen. Because the primer sequences were not indicated, however, it is unclear which form of aromatase was amplified in that study. It is possible that the post-transcriptional splicing to yield the short form of aromatase is regulated by E2, which could explain E2 regulation in Iivonen et al. [40].

### Regulation of aromatase expression by factors other than gonadal hormones

While a majority of studies in the brain have focused on hormonal modulation, the structure of the aromatase gene indicates multiple points of regulation, and aromatase is regulated by non-hormonal factors in peripheral tissues. For example, tissue-specific expression of aromatase is regulated through different promoter regions and alternative splicing. Cloning and structural characterization of the aromatase gene showed that the coding region spans 9 exons beginning with exon 2 [41]–[43]. There are multiple potential variants of exon 1, based on alternative transcription initiation sites, which are spliced into the 5′-untranslated region (5′-UTR) [1]; however, the coding region, and thus the protein expressed, is the same. Although each of the alternative aromatase transcripts has been isolated from brains of both male and female mice, one transcript generates more than 85% of all aromatase transcripts in brains of both sexes [44]. Whether differences in the 5′-UTR lead to differential regulation of aromatase at the translational level has not yet been investigated in the brain. Interestingly, the promoter regions of the aromatase gene contain a wide array of responsive elements leading to differential regulation among tissues. For example, aromatase expression in the ovary is regulated primarily by cAMP [45], in the placenta by retinoids [46], in adipose tissue by cytokines such as IL-6 and IL-11, as well as TNF-alpha [45], and in osteoblasts by glucocorticoids [47]. It is possible that aromatase expression in the brain is also regulated by one or several of these factors.

We found that levels of long-form aromatase in non-reproductive brain regions such as the hippocampus were similar between males and females and not affected by gonadal/hormonal status. This is useful in the context of acute E2 modulation of synaptic transmission in the hippocampus, as it eliminates sex and gonadal/hormonal status as factors that could influence the supply of locally synthesized estrogens through differential aromatase expression. Future studies will need to investigate non-hormonal factors that regulate aromatase expression and/or activity in the hippocampus and that could therefore alter the capacity of the hippocampus to synthesize estrogens. Identifying non-hormonal factors that regulate aromatase in non-reproductive brain regions such as the hippocampus will be critical to understanding the physiological role of acute E2 modulation of synaptic physiology for non-reproductive brain functions such as affective behaviors and learning and memory.
